# Study protocol for the Fex-Can Childhood project: An observational study and a randomized controlled trial focusing on sexual dysfunction and fertility-related distress in young adult survivors of childhood cancer

**DOI:** 10.1097/MD.0000000000019919

**Published:** 2020-07-10

**Authors:** Lisa Ljungman, Poorna Anandavadivelan, Kirsi Jahnukainen, Claudia Lampic, Lena Wettergren

**Affiliations:** a Department of Women's and Children's Health, Karolinska Institutet, Stockholm, Sweden; b Division of Haematology-Oncology and Stem Cell Transplantation, Children's Hospital, University of Helsinki, Helsinki University Central Hospital, Helsinki, Finland; c NORDFERTIL Research Lab Stockholm, Childhood Cancer Research Unit, Department of Women's and Children's Health, Karolinska Institutet and University Hospital, Stockholm, Sweden; d Department of Public Health and Caring Sciences, Uppsala University, Uppsala, Sweden.

**Keywords:** childhood cancer, fertility-related distress, intervention, observational study, randomized controlled trial, sexual function, survivors, young adults

## Abstract

**Background:**

This study protocol describes the Fex-Can Childhood project, comprising two studies: The Fex-Can Childhood observational study (OS) and the Fex-Can Childhood randomized controlled trial (RCT). The Fex-Can Childhood OS aims to determine the prevalence and predictors of sexual dysfunction and fertility-related distress in young adult childhood cancer survivors (aged 19–40) compared to an age matched comparison group; the Fex-Can Childhood RCT will evaluate the effect of a web-based psycho-educational intervention (Fex-Can intervention) on sexual dysfunction and fertility-related distress.

**Methods:**

The Fex-Can Childhood OS will have a population-based cross-sectional design. All individuals treated for childhood cancer in Sweden at the age of 0 to 17 years (current age 19–40) will be identified through the National Quality Registry for Childhood Cancer. Established self-reported instruments will be used to measure sexual function, fertility-related distress, body image, anxiety and depression, and health-related quality of life. Self-efficacy related to sexual function and fertility, and fertility-related knowledge, will be assessed by study-specific measures. Clinical variables will be collected from the registry. Results will be compared to an age-matched comparison group from the general population.

Participants in the Fex-Can Childhood OS who report a high level of sexual dysfunction and/or fertility-related distress will be invited to participate in the RCT. The Fex-Can intervention comprises two programs: The Fex-Can Sex and the Fex-Can Fertility targeting sexual dysfunction and fertility-related distress, respectively. The control condition will be a wait-list. Sexual function and fertility-related distress will be the primary outcomes. The secondary outcomes include body image, anxiety and depression, health-related quality of life and self-efficacy related to sexual function and fertility. Post- and follow-up assessments will be conducted directly after end of intervention (primary end point), at 3 months and 6 months after end of intervention. Additionally, a process-evaluation including study-specific items and a qualitative interview will be conducted.

**Discussion:**

The Fex-Can Childhood project will advance knowledge in the areas of sexual function and fertility-related distress among young adult survivors of childhood cancer. If the Fex-Can intervention proves to be efficacious, steps will be taken to implement it in the follow-up care provided to this population.

## Abstract 2

Supplemental Digital Content is available in the text

## 1 Introduction

### 1.1 Background and rationale

Due to improvements in treatments for childhood cancer in the recent decades, survival rates have increased dramatically and are now reaching 80% to 85% in most developed countries. There are currently about 11,000 survivors of childhood cancer in Sweden, and a substantial proportion of them are young adults (19–40 years).^[1]^ The growing number of survivors has led to increased attention to the late effects of childhood cancer, including impaired sexual function and infertility. Sexual and reproductive health are salient issues during young adulthood and thus strong determinants of the quality of long-term survivorship.^[2]^

Among young adult survivors of childhood cancer approximately half of the women and one third of the men report problems in one or more areas of sexual functioning.^[3–7]^ Problems include low sexual interest, impaired ability to get aroused and low satisfaction with sex life.^[8]^ Survivors of brain tumors and survivors who received their cancer diagnosis during adolescence seem to be at particular risk of poor sexual function.^[6,8]^ Disruptions in normal pubertal development, premature ovarian failure, and/or the burden of medical late effects have been suggested as mechanisms involved in these problems.^[4,9,10]^ Additionally, psychological factors such as anxiety, depression and negative body image have been associated to sexual dysfunction following cancer in young adults.^[11,12]^ Still, due to a paucity of large-scale methodologically rigid studies, firm knowledge about prevalence rates and the factors causing sexual problems in survivors of childhood cancer is lacking.

Cancer therapy can have adverse effects on the reproductive health in both male and female childhood cancer survivors. The prevalence of gonadal dysfunction has been reported to be as high as 66% among males and 11% among female survivors (median age of 32 years) who were exposed to abdominal/pelvic radiation and/or to alkylating agents during early childhood.^[13,14]^ Given the age-related natural decline in follicular reserve, older age at treatment exposure has also been described as an additional risk factor for gonadal dysfunction in females.^[15]^ Childhood cancer survivors thus face reduced fertility compared to their siblings^[15–18]^ and to the general population.^[19,20]^ Despite the possible threat of sub-fertility/infertility following treatment for childhood cancer, many survivors are unaware of their fertility status.^[21–25]^ Worry about not being able to have a child can be particularly challenging and lead to distress described as “fertility-related distress.”^[26]^ Health of the future biological children, own health in the future, and pregnancy-induced cancer recurrence are seemingly major concerns for survivors.^[23,27–29]^ However, both qualitative and population-based studies report that survivors’ perception of own fertility status are not aligned with their actual fertility status.^[30–32]^ It is therefore likely that misperceptions about fertility lead to unnecessary and inappropriate worry regarding fertility.^[33]^ Moreover, population-based studies with accurate estimates of the prevalence of fertility-related distress in childhood cancer survivorship are lacking.

In order to advance, this field of knowledge, there is a need to establish prevalence rates of sexual dysfunction and of fertility-related distress in young adults treated for cancer during their childhood. To reach such aim, large-scale and methodologically rigorous studies investigating these issues in both male and female survivors are needed. Also, reliable comparisons with the general population and investigations of the association between these issues and different cancer types, treatment intensity, and age at diagnosis should be made. Furthermore, even though sexual problems and fertility-related distress have been reported among young adult survivors of childhood cancer in numerous previous studies, there is a lack of specific interventions targeting these issues. Web-based or e-Health interventions have been established to be efficacious in terms of improvement of quality of life in a variety of somatic populations.^[34–37]^ There are a few studies that have focused on the effectiveness of web-based interventions to overcome sexual and reproductive problems following cancer. One such intervention showed improved social and physical functioning and fertility-related knowledge in young adults in the aftermath of cancer.^[38]^ Another intervention aimed to decrease sexual problems in female breast cancer survivors and also demonstrated positive effects over time.^[39]^ Still, to date, no web-based intervention with the aim to reduce sexual problems and fertility-related distress has been developed and tested in young adult survivors of childhood cancer.

With the above concerns in consideration the project Fertility and Sexuality following Cancer (Fex-Can) was launched. The overall aim of the Fex-Can project is to improve sexual and reproductive health in young adults who have been treated for cancer. Within this project, a web-based intervention (Fex-Can intervention) has been developed in close cooperation with young adults with a cancer experience.^[40]^ The Fex-Can intervention is a web-based psychoeducational intervention consisting of two programs; the Fex-Can Sex and the Fex-Can Fertility, targeting sexual dysfunction and fertility-related distress, respectively. The Fex-Can intervention has been evaluated as to its feasibility with satisfactory results.^[41]^ Besides the Fex-Can Childhood project described in this protocol, a sister project focusing on sexual problems and fertility-related distress in young adults recently diagnosed with cancer is currently running, as described in two separate protocols.^[42,43]^

The Fex-Can Childhood project targets young adult survivors of childhood cancer (cancer diagnosis at the age of 0–17 and currently aged 19–40) and consists of two studies:

1. A population-based observational study determining sexual dysfunction and fertility-related distress in this population; the Fex-Can Childhood observational study (Fex-Can Childhood OS), and
2. A randomized controlled trial (RCT) evaluating the Fex-Can intervention; the Fex-Can Childhood RCT.

The present protocol describes the whole Fex-Can Childhood project including both the Fex-Can Childhood OS and the Fex-Can Childhood RCT.

### 1.2 Objectives

The aim of the Fex-Can Childhood OS is to determine the prevalence and predictors of sexual dysfunction and fertility-related distress in young adult childhood cancer survivors (aged 19–40) compared to an age matched comparison group. The aim of the Fex-Can Childhood RCT is to evaluate whether the Fex-Can intervention is effective in terms of reduction of sexual dysfunction and fertility-related distress in young adult survivors of childhood cancer. An additional aim of the Fex-Can Childhood RCT is to test the effect of the Fex-Can intervention on the secondary outcomes, that is, health-related quality of life, symptoms of anxiety and depression, body image, fertility knowledge, and self-efficacy related to sexuality and fertility.

## 2 Methods

### 2.1 Design

**Figure 1:**
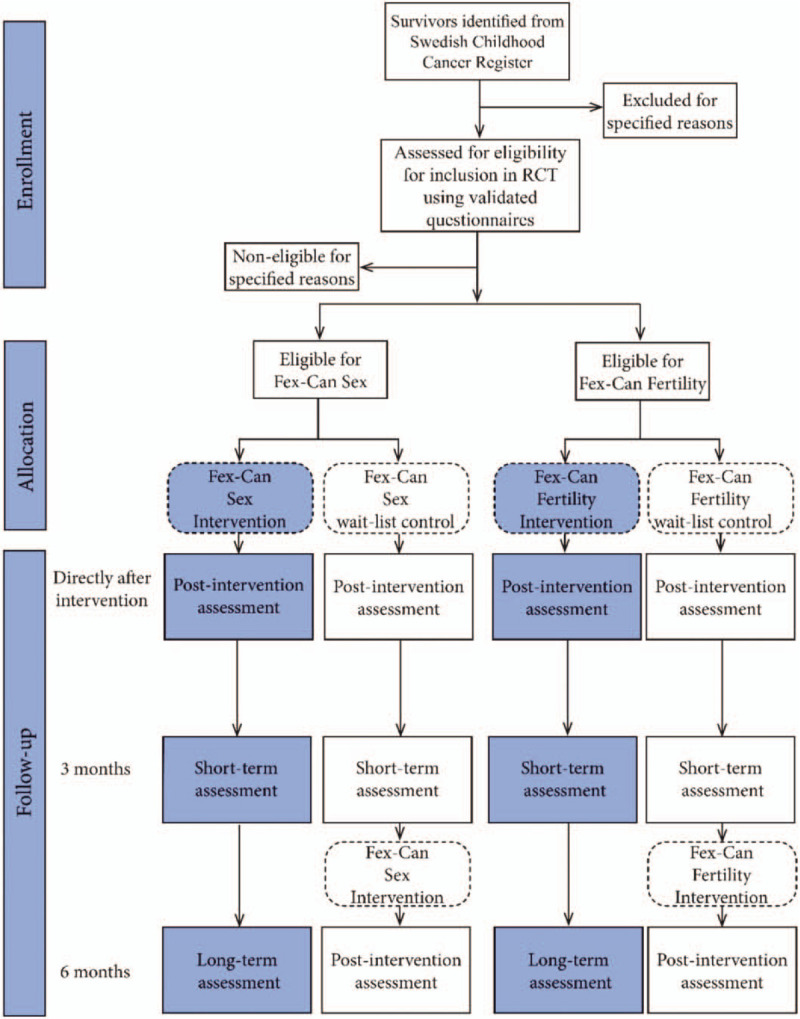
Consort-flow chart for the Fex-Can Childhood RCT

The Fex-Can Childhood project started January 2019 and is expected to run till November 2020. The Fex-Can Childhood OS will have a population-based cross-sectional design, including an age matched comparison group. The Fex-Can Childhood RCT will test the effect of the Fex-Can intervention by using an RCT with a superiority design and a 1:1 allocation ratio. The active group will receive the Fex-Can intervention for 12 weeks (either the Fex-Can Sex or the Fex-Can Fertility version of the program) and the control group will be on a wait-list. The effect of the Fex-Can intervention will be evaluated directly after end of the program (primary endpoint), and at 3-month follow-up. The control group will receive access to the intervention after the 3-month follow-up assessment. An additional follow-up assessment will be conducted at 6 months after end of the program which will be used to analyze sustainment of a potential effect for the intervention group. To achieve a deeper understanding of the participants’, use of the intervention, and of the role of the program in bringing about a possible change in the selected outcomes, a process-evaluation including survey questions and qualitative interviews will also be conducted. See Figure 1 for flow diagram of the Fex-Can Childhood RCT.

### 2.2 Study setting and recruitment

#### 2.2.1 Fex-Can Childhood OS

- Cancer survivor group

Participants will be identified using the National Quality Registry for Childhood Cancer which is a quality registry of all children diagnosed with cancer or brain tumors who are treated at pediatric oncology centers in Sweden.^[44]^ At present, the registry includes ∼4500 survivors of childhood cancer in the age-span 19 to 40 years. A letter with study information will be sent to all individuals in the registry matching the inclusion criteria (see below), emphasizing that participation is voluntary and that non-participation will not affect potential care. On acceptance to participate, self-administered questionnaires will be collected by paper or web. Two reminders will be sent to non-responders. Participants will be rewarded with two cinema tickets for participating in the study.

- Comparison group

A comparison group consisting of a random sample of 2000 individuals (1000 women and 1000 men) drawn from the Swedish population register (SPAR) have been approached regarding study participation. The self-administered questionnaires (same as for the cancer group besides cancer specific items which were edited out), together with a letter with information about the study, including the voluntary nature of participation, was sent to potential participants. As for the cancer group, it was optional to complete the survey via paper or web, and two reminders were sent to non-responders. The comparison group was offered the same compensation for participation as the cancer group, that is, two cinema tickets. The comparison group data was collected during 2018.

#### 2.2.2 Fex-Can Childhood RCT

Participants in the Fex-Can Childhood OS study (cancer survivor group) who report sexual dysfunction or fertility-related distress will be invited to participate in the Fex-Can Childhood RCT (see criteria below). Invitation will be sent via post and individuals who accept participation will be allocated to either the Fex-Can Sex or Fex-Can Fertility program according to the primary issues reported by them, that is, the Fex-Can Sex for those reporting high levels of sexual dysfunction and Fex-Can fertility for those reporting high levels of fertility-related concerns.

The assessment in the Fex-Can Childhood OS will be used as baseline assessment in the Fex-Can Childhood RCT. Post- and follow-up assessments will be collected directly after end of intervention, at 3 months after end of intervention (short-term follow-up), and at 6 months after end of the intervention (long-term follow-up). Participants in the wait-list control group will receive the intervention after completion of the short-term follow-up assessment. Directly after the intervention they will also complete the post-intervention assessment.

### 2.3 Eligibility criteria

#### 2.3.1 Inclusion criteria Fex-Can Childhood OS

- Cancer survivor group

1. Individuals diagnosed with malignant disease at the age of 0 to 17 years and registered in the National Quality Registry for Childhood Cancer.
2. Age 19 to 40 at the time of enrollment and registered as residents in Sweden.

- Comparison group

1. Age 19 to 40 at the time of enrollment (matching the age of the cancer group) and registered as residents in Sweden.

#### 2.3.2 Inclusion criteria Fex-Can Childhood RCT

Participating in the Fex-Can Childhood OS study (cancer survivor group) and reporting a high level of sexual dysfunction and/or fertility-related distress. In order to be eligible to be enrolled into the Fex-Can Sex program participants should report sexual dysfunction (defined as 0.5 SD from the population mean) in at least one of the selected domains (for specification of domains, see below under Primary outcomes) of the Patient-Reported Outcomes Measurement Information System Sexual Function and Satisfaction measure version 2.0 (SexFS version 2.0), or report not having had sex due to cancer-related reasons.^[45]^ To be eligible to be enrolled in the Fex-Can Fertility program, individuals should report fertility distress (defined as a mean score >4) in at least one dimension of the Reproductive Concerns After Cancer (RCAC)^[46]^ (for further description of these assessments, see Section 4.3.3).

#### 2.3.3 Exclusion criteria Fex-Can Childhood OS/RCT

1. Individuals who are unable to read/write in the Swedish language.
2. Individuals who report poor health and/or substantial cognitive impairment that prevent completion of the survey and/or participation in the intervention.

### 2.4 Intervention

The Fex-Can Intervention is a web-based self-help intervention that comprises two programs: The Fex-Can Sex and the Fex-Can Fertility targeting sexual dysfunction and fertility-related distress, respectively. The intervention has originally been developed together with young adult survivors of cancer as research partners (described in detail elsewhere using a co-creative long-term collaboration).^[40]^ The feasibility assessment of the intervention has provided satisfying results, rendering it suitable for young persons with cancer in terms of demand, acceptability, preliminary efficacy, and functionality.^[8]^ Both the Fex-Can Sex and the Fex-Can Fertility programs are structured in six modules which will be delivered over a period of 12 consecutive weeks. A detailed description of the modules and their content has been presented elsewhere.^[41]^ Briefly, the modules comprise educational and behavior modification content, exercises, illustrations and quizzes. For the Fex-Can Childhood RCT, all contents of the modules have been adapted to be suitable for participants previously diagnosed with childhood cancer with respect to the age of diagnosis (0–17 years), various types of childhood cancer diagnoses and common treatment modalities. The program has been created bearing in mind the likelihood that some of the participants may not remember having experienced cancer and its treatment. Great care has also been taken to develop the intervention to fit participants irrespective of sexual orientation, if they have a partner or not, and if they have or have not had sex. Additionally, the modules include video vignettes of young adult survivors of childhood cancer describing their experiences regarding the topics covered. The aim of the exercises is to increase sexual pleasure and function (Fex-Can Sex), and counteract worries related to threatened or lost fertility and on managing parenthood (Fex-Can Fertility). The intervention also includes an online-moderated discussion forum, that is, join between the two programs. The intervention will be delivered on a platform with a responsive design suitable to be used on computers, tablets, and smartphones.

#### 2.4.1 Compliance

In order to achieve a high compliance to the intervention, participants will receive text/e-mail notifications at the opening of each of the six modules. Compliance will be evaluated through the log function in the web platform that records the log-in information of all participants and also their usage of different program functions. Furthermore, the post-intervention assessment (directly after end of program) will include questions about the participants’ use of different program features.

#### 2.4.2 Concomitant care

Concomitant care and treatment for sexual dysfunction and/or fertility-related distress will not be inhibited by study participation. Information about the use of such care will be collected in the post- and follow-up assessments.

### 2.5 Measurements

All standardized measures will be used and analyzed in line with the guidelines provided in their respective manuals. The questionnaire battery has been tested in two groups of young women and men with cancer and showed high acceptance level.^[47,48]^ See Table 1 for a presentation of the administration of instruments.

**Table 1:**
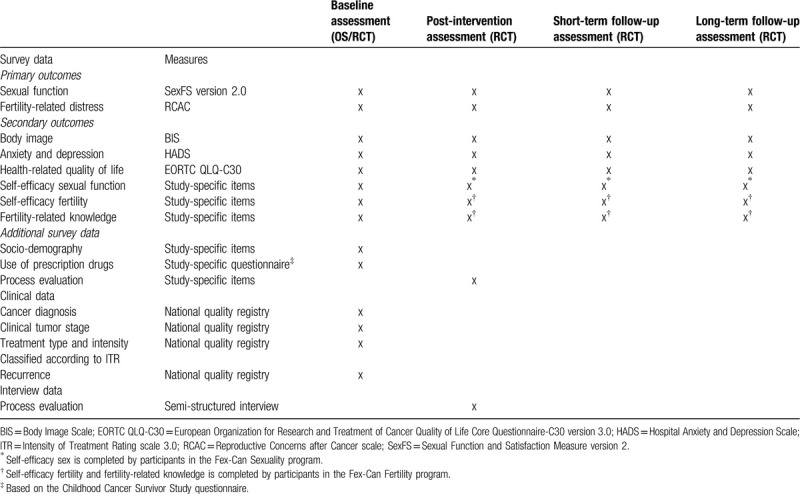
Timeline for Fex-Can Childhood OS/RCT.

### 2.6 Primary outcomes

#### 2.6.1 Fex-Can Childhood OS/RCT

- Sexual function

Sexual function will be assessed using the SexFS version 2.0. The SexFS version 2.0 is a self-rated measurement that consists of a set of domains that represents different areas of sexual function and satisfaction. For the present study the following domains will be selected for women: Vaginal lubrication, Vaginal discomfort, Vulvar discomfort—clitoral, and Vulvar discomfort—labial. For males, the domain Erectile function will be selected. Additionally, four gender-neutral domains will be included for all participants: Interest in sexual activity, Orgasm—ability, Orgasm—pleasure, and Satisfaction with sex life. For most items responses in the SexFS version 2.0 are given with five options from, for example, “never” to “always.” Item responses in each domain are used to calculate domain scores which are transformed to a T-score metric where 50 represents the mean for the American general population (standard deviation = 10).^[45]^ In addition to the domains, items assessing bother related to sexual function and screener items assessing sexual activity will be included.

The SexFS has shown adequate content, construct and known-groups validity as well as test-retest reliability.^[45,49]^ The selected items and domains of the SexFS version 2.0 have been translated into Swedish and linguistically validated in accordance with the procedure developed by FACITrans and PROMIS.^[50]^

- Fertility-related distress

Fertility-related distress will be assessed using the RCAC. The RCAC is a self-rated multidimensional scale, measuring a range of fertility and parenthood concerns, originally developed and evaluated for young adult female cancer survivors (age 18–35 years).^[46]^ There are 18 items in the scale, the responses for which are scored on a five-point scale (ranging from 1 = strongly disagree to 5 = strongly agree). The RCAC includes six dimensions: Fertility potential, Partner disclosure, Child's health, Personal health, Acceptance, and Becoming pregnant/Achieving pregnancy (each dimension consists of three items). In each dimension, a high level of reproductive concerns is defined as a mean score ≥4. The RCAC has demonstrated satisfactory internal consistency and construct validity.^[46,51,52]^ The scale has independently been translated into Swedish by two bilingual researchers, and has been evaluated by one bilingual panel (n = 4), one lay panel (n = 7), and one patient panel (n = 8), as well as by cognitive interviews with 3 young patients with cancer before the launch of the Fex-Can project.

#### 2.6.2 Fex-Can Childhood RCT

The primary outcome in the Fex-Can Sex program will be the total score (continuous measure) in the domain ‘Satisfaction with sex life’ whereas the overall mean score of the RCAC will be used to evaluate effect of the Fex-Can Fertility program.

### 2.7 Secondary outcomes

#### 2.7.1 Fex-Can Childhood OS/RCT

The secondary outcomes will be fertility-related knowledge (the Fex-Can Fertility); body image; health-related quality of life; symptoms of anxiety and depression; and self-efficacy related to sexuality and fertility (the Fex-Can Sex and the Fex-Can Fertility, respectively). In addition, secondary outcomes will include all the domains of the SexFS version 2.0 besides “Satisfaction with sex life” (used as primary outcome), the items assessing bother related to sexual function, and screener items assessing sexual activity. Also, the specific dimensions of RCAC will be used as secondary outcomes.

- Fertility-related knowledge

A study-specific questionnaire will be used to assess fertility-related knowledge. The questionnaire includes 10 items measuring perceived level of knowledge about general and cancer-related fertility issues. For males, there are totally 9 items with the exception of one item regarding the effect of the cancer/cancer treatment on pregnancy and child birth. Responses are provided on a four-point scale ranging from 1 = disagree completely to 4 = agree completely. Examples of items are: “I have good knowledge regarding the effect of age on the reproductive ability” and “I have good knowledge regarding the effect of cancer and cancer treatments on pregnancy and delivery.” Total mean scores will be calculated with higher scores indicating higher levels of perceived fertility-related knowledge.

- Body image

The Body Image Scale (BIS) will be used to assess body image discomfort associated with cancer and cancer treatment.^[53]^ The BIS contains 10 items which are scored from 0 to 3 with higher scores indicating a more negative body image. The BIS has shown high test–retest reliability and good internal consistency in cancer patients.^[53]^

- Health-related quality of life

The QLQ-C30 (version 3.0), developed by the European Organization for Research and Treatment of Cancer (EORTC), will be used to assess health related quality of life. The QLQ-C30 (version 3.0), is a 30-item questionnaire developed to assess the general aspects of quality of life and common symptoms specific to cancer patients.^[54,55]^ It includes five functional scales (physical, role, emotional, cognitive, and social), three symptom scales (fatigue, nausea/vomiting, and pain), a global health status scale, and six single items (dyspnea, insomnia, appetite loss, constipation, diarrhea, and financial impact). The questionnaire has exhibited good psychometric properties in cancer populations.^[13,15]^ The summary score for the QLQ-30 will be used according to the EORTC QLQ-C30 Scoring Manual (3rd Edition) (2001).^[55]^

- Anxiety and depression

The Hospital Anxiety and Depression scale (HADS) will be used to assess anxiety and depression.^[56]^ The HADS encompasses two subscales with 7 items each, measuring symptoms of anxiety and depression, respectively. Both subscales have shown satisfactory internal consistency and good to very good concurrent validity.^[57]^

- Self-efficacy

Self-efficacy will be evaluated using a study-specific questionnaire assessing the participant's confidence in one's own ability to handle situations, thoughts and emotions related to the threat of infertility (6 items) and sexuality (6 items). For example, “I feel confident that I have a positive attitude about my sex life” (Fex-Can Sex) and “I feel confident that I can deal with meeting friends or relatives expecting a child” (Fex-Can Childhood Fertility). Total mean scores will be with higher scores indicating higher levels of self-efficacy related to sexuality and fertility, respectively.

### 2.8 Additional survey data

In the survey, questions regarding socio-demographics will also be included. Additionally, the use of prescription drugs over the last 2 years will be assessed by use of a study-specific questionnaire. It is a shortened version of a comprehensive survey developed for use in survivors of childhood acute myeloid leukemia in the Nordic countries^[58,59]^ originally inspired by the Childhood Cancer Survivor Study questionnaire.^[60]^ The items in the questionnaire were adapted for assessment of prescription drug usage in childhood cancer survivors included in the Fex-Can Childhood project.

### 2.9 Administration of instruments

The instruments will be administered in the following order at all assessments: BIS; RCAC; Self-efficacy Fertility; Fertility-related knowledge; Self-efficacy Sexuality; SexFS version 2.0; HADS; EORTC QLQ-30 version 3.0; and use of prescription drugs.

### 2.10 Clinical data

#### 2.10.1 Fex-Can Childhood OS/RCT

Following formal consent from the National Quality Registry for Childhood Cancer, clinical data will be obtained. The data will include cancer diagnosis and clinical stage, date of diagnosis, treatment type and intensity, and recurrence or new cancer diagnosis after treatment. Treatment intensity will be categorized in accordance with the Intensity of Treatment Rating scale (ITR-3.0).^[61]^ Clinical variables will be selected in close collaboration with a clinician (KJ) with vast expertise in pediatric oncology and a representative from the National Quality Registry for Childhood Cancer in Sweden.

### 2.11 Process evaluation

#### 2.11.1 Fex-Can Childhood RCT

- Post-intervention survey

In addition to the primary and secondary outcomes, the post-intervention survey will include questions regarding adherence to the program, use of the different features of the program, and perception of change in sexual problems and/or fertility-related distress. These questions will be administered to the participants belonging to the intervention group and will encompass 13 study-specific items. Participants will also be requested to comment on the content and features of the intervention. Responses will be provided on a four-point scale ranging from “Disagree completely” to “Agree completely.” Additionally, participants will be requested to score their present levels of problems (concerning sex life or fertility-related distress) in comparison to the level before they entered the Fex-Can Childhood intervention. Responses will be given on a 7-point scale, ranging from “Much improved” to “Much worsened” with the midpoint “No change.”

#### 2.11.2 Semi-structured interview

Semi-structured interviews will be conducted with a purposive sample of ∼30 participants in the intervention group in order to achieve a deeper understanding of the intervention usage, and the role of the program in bringing about a change in the outcomes. The interviews will be performed via telephone, shortly after completion of the intervention. Questions will explore the participants’ view of their sexual problems or fertility-related distress and if/how these problems have changed during the course of the intervention. Participants will also be asked to describe their experiences of participating in the intervention and their use of the different program functions. The interviews will be digitally recorded, transcribed verbatim and will be analyzed using qualitative content analysis.^[62]^

### 2.12 Participant timeline

See Table 1 for study timeline.

### 2.13 Sample size

All eligible individuals registered in the National Quality Registry for Childhood Cancer and matching the inclusion criteria for the Fex-Can Childhood OS-study (∼4500) will be approached and invited to participate. With an estimated response rate of 60% the sample size (n = 2700) will be large enough to analyze clinical and sociodemographic predictors of the sexual dysfunction and fertility-related distress. With regards to the Fex-Can RCT we expect ∼50% of the participants in the Fex-Can OS study to rate high levels of sexual problems or fertility-related distress (n = 1350). These individuals will be invited to participate in the RCT. In order to detect a statistically significant group difference with a power of 80%, estimating medium effect size (0.5) and α = 0.05, a total of 128 completers (post-intervention assessment) will be required in each version of the program in the RCT. We expect ∼30% of the individuals who are invited to the RCT to agree to participate (n = 405). With an estimated attrition rate of 15% at the post-intervention assessment we will reach well over 256 completers and achieve adequate statistical power in the Fex-Can RCT.

### 2.14 Randomization

Participants will be randomly assigned to either intervention-group or wait-list control-group with an allocation ratio of 1:1. Participants will be allocated in blocks stratified by sex and diagnosis (leukemia/lymphoma; brain tumor; solid tumor). The randomization will be carried out separately for the two programs of the RCT (Fex-Can Childhood Sex and Fex-Can Childhood Fertility). In the event that participants’ eligibility criteria are met for both programs, an evaluation of individual scores will be performed based on severity of reported problems by a registered psychologist and a registered nurse specialized in psychosocial oncology.

In order to minimize bias, the computer-generated randomization sequence will be created by a statistician not directly involved in the management of the trial and the information regarding the series of random numbers will be kept unknown to the investigators.

### 2.15 Blinding

Due to the nature of the intervention, it is not possible to conceal the participants’ group allocation to the researchers involved in providing and monitoring the intervention and data collection. Hence, full blinding is infeasible during the intervention in the present study, however, participants’ allocation to intervention or control groups will be masked in the data set available for researchers during data analysis.

### 2.16 Statistical methods

#### 2.16.1 Fex-Can Childhood OS

All data analyses will be performed and reported in accordance with STROBE^[63]^ for observational studies. With the exception of descriptive statistics and visualizations, multiple regression models will be the main class of statistical method used to analyze prevalence and predictors of the outcomes. Missing data will be investigated using descriptive statistics and significance testing.

#### 2.16.2 Fex-Can Childhood RCT

All data analyses will be performed and reported in line with the SPIRIT-PRO Extension for randomized clinical trials.^[64]^ The statistical analyses will primarily aim at comparing the intervention with the control group directly after end of program. The 3-month follow-up assessment will be used as secondary end-point comparing the intervention group and the control group. In addition, sustainment of a potential effect for the intervention group at 6 months (long-term assessment) will be evaluated. Missing data will be investigated using descriptive statistics and significance testing. Intention-to-treat analyses will be applied for the primary outcomes in the RCT.

All statistical analyses will be conducted by external statisticians, who are not aware of the study participants’ group allocation. SPSS Statistics version 26 (IBM Corp., Armonk, NY) will be used for data management and statistical analyses.

### 2.17 Adverse effects

In order to assess adverse effects for participants in the Fex-Can Childhood RCT, the number of participants reporting possible worsening of symptoms in the primary or secondary outcomes will be evaluated. Furthermore, participants will be asked to specify in the post-intervention survey if they have experienced any worsening of symptoms. Additionally, during the semi-structured post-intervention interview, participants will be asked to describe possible adverse effects experienced.

### 2.18 Ethics and dissemination

#### 2.18.1 Research ethics approval

Ethical approval has been granted by the Regional Ethical Review Board in Stockholm, Sweden (Dnr: 2015/1609-31; 2018/2688-32; 2019/01066; 2019/04603).

#### 2.18.2 Confidentiality

A unique code number will be assigned to all participants and indicated on the survey. The code key will be maintained separately from the research data and will only be accessible by members of the research team. Participants will access the web portal by using an alias of their own choice. The researchers will be able to connect participants’ alias to the code number only at the stage of analyzing data. All data will be handled and stored in accordance to the EU General Data Protection Regulation (GDPR).

#### 2.18.3 Dissemination policy

The results from the trial will be communicated to the scientific, clinical and patient communities through publications in scientific peer-reviewed open-access journals and presentations at international clinical and scientific conferences and in other contexts.

## 3 Discussion

Several studies of childhood cancer survivors demonstrate impairment in sexual and reproductive functions.^[15,65,66]^ This include different types of sexual dysfunctions, negative impact on intimate relations, and the emotional consequences of living with threatened or actual infertility. There is, however, a lack of large-scale methodologically rigid studies on the prevalence of these issues, and of the factors associated to them. Furthermore, there are no studies evaluating interventions to decrease sexual problems and fertility-related distress in adult survivors of childhood cancer. The Fex-Can Childhood project includes two studies aiming to fill these gaps in the previous literature. The observational study, the Fex-Can Childhood OS, will determine occurrence and predictors of sexual problems and fertility-related distress. Fex-Can Childhood RCT on the other hand will evaluate the effect of a web-based self-help intervention that aims to alleviate these issues among childhood cancer survivors.

There are several strengths of the design of the studies in this project. First, the Fex-Can Childhood OS will have a population-based inclusion procedure using the National Quality Registry for Childhood Cancer, which allows for identification of the whole population of young adult childhood cancer survivors in Sweden. Also, the use of clinical data from the registry will provide high quality clinical variables consolidated from the various registries for childhood cancer diagnosis.^[44,67]^ Furthermore, a strength of the study is the collection of comparison data from the general population of the same age using the same outcome measurements. This will provide reliable comparison data and thus, reliable estimates of prevalence in the target population. Lastly, strength of the study is the use of established instruments that have undergone psychometric testing instruments of sexual function and fertility-related distress, as well as of secondary outcomes.

With regards to the Fex-Can Childhood RCT, a main strength is the RCT-design which is the gold standard in clinical research. Another major strength is the two parallel-arm-designs, which will allow evaluation of both versions of the intervention; the Fex-Can Sex (targeting sexual problems) and the Fex-Can Fertility (targeting fertility-related distress). In addition, the assessment of several secondary outcomes will investigate additional effects of the intervention as well as potential interactions with other processes related to the outcomes, which will enhance the clinical relevance of conclusions. Lastly, by providing the intervention to the wait-list control group after end of the short-term follow-up assessment, all participants will be offered the intervention which may increase motivation to participate in the trial, and thus minimize selection bias.

Some potential limitations of the project should also be mentioned. For the Fex-Can Childhood RCT a potential limitation is the lack of certainty with regards to prevalence rates of sexual problems and fertility-related distress in this population since these are key determinants of inclusion rate. Prevalence rates will impact the number of participants that can be invited to participate in the RCT, and the number that will be included, and thus will affect the possibility to reach adequate statistical power in the study. In addition, the cut-off values selected for inclusion have not been evaluated previously and the clinical relevance of sexual problems and fertility-related distress at levels above these cut-offs are yet to be determined.

In conclusion, the present study protocol describes a population-based observational study investigating sexual problems and fertility-related distress in young adult childhood cancer survivors and the clinical trial of the Fex-Can intervention; the first web-based intervention targeting sexual problems and fertility-related distress in this population. The results from these studies will have significant clinical implications by both determining the prevalence of sexual problems and fertility-related distress in the aftermath of childhood cancer, and by the potential to provide an effective intervention aiming to alleviate these issues. In the event that the Fex-Can intervention proves to be efficacious, steps will be taken to implement it in routine care for young adult childhood cancer survivors. Together these two studies that constitute the Fex-Can Childhood project, will advance knowledge in the area and has the potential to improve care provided to childhood cancer survivors in the future.

See Appendix.

## Author contributions

**Conceptualization:** Claudia Lampic, Lena Wettergren.

**Data curation:** Lisa Ljungman, Poorna Anandavadivelan, Kirsi Jahnukainen, Claudia Lampic, Lena Wettergren.

**Funding acquisition:** Claudia Lampic, Lena Wettergren.

**Methodology:** Lisa Ljungman, Poorna Anandavadivelan, Kirsi Jahnukainen, Claudia Lampic, Lena Wettergren.

**Project administration:** Claudia Lampic, Lena Wettergren.

**Resources:** Claudia Lampic, Lena Wettergren.

**Supervision:** Claudia Lampic, Lena Wettergren.

**Visualization:** Lena Wettergren, Claudia Lampic.

**Writing – original draft:** Lisa Ljungman, Poorna Anandavadivelan.

**Writing – review & editing:** Lisa Ljungman, Poorna Anandavadivelan, Kirsi Jahnukainen, Claudia Lampic, Lena Wettergren.

Lena Wettergren: ORCID ID: 0000-0003-1279-2191.

## Supplementary Material

Supplemental Digital Content
