# Supplementary material for: Study protocol for the Fex-Can Childhood project: An observational study and a randomized controlled trial focusing on sexual dysfunction and fertility-related distress in young adult survivors of childhood cancer
Source: Medicine (Baltimore). 2020 Jul 10;99(28):e19919. doi: 10.1097/MD.0000000000019919 (PMC7360293; doi:10.1097/MD.0000000000019919)
Supplement: Supplemental Digital Content [file medi-99-e19919-s001.docx]

Items from the World Health Organization Trial Registration Data Set

| **Data category** | **Information** |
| --- | --- |
| Primary registry and trial identifying number | ISRCTN, ISRCTN33081791. |
| Date of registration in primary registry | 27 November 2019 |
| Secondary identifying numbers | http://www.isrctn.com/ISRCTN33081791 |
| Source(s) of monetary or material support | Karolinska Institutet |
| Primary sponsor | The Cancer Research Foundations of Radiumhemmet (grant number 161272); the Swedish Cancer Society (CAN 2013/886 and CAN 2016/615); the Swedish Childhood Cancer Foundation (TJ2014-0050, TJ2019-0045, PR2014-0177); the Vårdal Foundation (2014-0098); the Swedish Research Council for Health, Working Life and Welfare (2014-4689); the Swedish Research Council (2017-01530); and the Karolinska Institutet Faculty Fonds (2-5586/2017). |
| Secondary sponsor(s) | NA |
| Contact for public queries | *Lena Wettergren*, PhD [+46 (0)8524836 50] [lena.wettergren@ki.se] *Claudia Lampic*, PhD [+ 46 (0)8524823 70] [claudia.lampic@ki.se] |
| Contact for scientific queries | *Lena Wettergren*, PhD Karolinska Institutet, Sweden |
| Public title | Sexual dysfunction and fertility-related distress in young adult survivors of childhood cancer. |
| Scientific title | The Fex-Can Childhood project – An observational study and a randomized controlled trial focusing on sexual dysfunction and fertility-related distress in young adult survivors of childhood cancer |
| Countries of recruitment | Sweden |
| Health condition(s) or problem(s) studied | Childhood cancer |
| Intervention(s) | The Fex-Can intervention consists of two programs, The Fex-Can Sex and the Fex-Can Fertility, targeting sexual dysfunction and fertility-related distress respectively. They are web-based self-help programs delivered over a period of 12 weeks. |
| Key inclusion and exclusion criteria | Ages eligible for study: 0-17 years at diagnosis Sexes eligible for study: both Accepts healthy volunteers: no Inclusion criteria: Individuals diagnosed with malignant disease at the age of 0-17 years and registered in the National Quality Registry for Childhood Cancer. Age 19-40 at the time of enrollment and registered as residents in Sweden. Additional inclusion criteria for Fex-Can Childhood RCT: Reporting high level of sexual dysfunction and/or fertility-related distress. Exclusion criteria: Individuals who are unable to read/write in the Swedish language and individuals who report poor health and/or substantial cognitive impairment that prevent completion of the survey and/or participation in the intervention. |
| Study type | Other Allocation: randomized Intervention model: parallel assignment in blocks stratified by sex and diagnosis. The process will be performed separately for the arms of the RCT (Fex-Can Sex and Fex-Can Fertility) Masking: not possible due to the nature of the intervention Primary purpose: Observational study and efficacy trial |
| Date of first enrolment | August 2019 (Observational study)/December 2019 (RCT) |
| Target sample size | 4500 (Observational study)  256 (Randomised clinical trial) |
| Recruitment status | Recruiting |
| Primary outcome(s) | The primary outcome for the Fex-Can Sex program is the score (continuous measure) of the domain ‘Satisfaction with sex life’, a subscale of the Patient-Reported Outcomes Measurement Information System® Sexual Function and Satisfaction measure. The primary outcome of the Fex-Can Fertility will be the summary score of the Reproductive Concerns After Cancer (RCAC). |
| Key secondary outcomes | Additional domains of sexual function, specific domains of reproductive concerns after cancer (RCAC) scale, body image assessed with the Body Image Scale (BIS); anxiety and depression assessed with the Hospital Anxiety and Depression scale (HADS); health-related quality of life measured with the EORTC QLQ-C30 (version 3.0); self-efficacy related to sexual function and fertility and fertility-related knowledge. |
